# Water Extract of *Angelica dahurica* Inhibits Osteoclast Differentiation and Bone Loss

**DOI:** 10.3390/ijms241914715

**Published:** 2023-09-28

**Authors:** Dong Ryun Gu, Hyun Yang, Seong Cheol Kim, Youn-Hwan Hwang, Hyunil Ha

**Affiliations:** 1 KM Convergence Research Division, Korea Institute of Oriental Medicine, Yuseong-daero 1672, Yuseong-gu, Daejeon 34054, Republic of Korea; mrwonsin@kiom.re.kr (D.R.G.);; 2 Korean Convergence Medicine Major KIOM, University of Science & Technology (UST), 1672 Yuseongdae-ro, Yuseong-gu, Daejeon 34054, Republic of Korea

**Keywords:** *Angelica dahurica*, osteoporosis, ovariectomy, osteoclast

## Abstract

*Angelica dahurica* radix has a long history of traditional use in China and Korea for treating headaches, cold-damp pain and skin diseases. Despite various pharmacological studies on *A. dahurica*, its impact on bones remains unclear. Hence, this study investigated the inhibitory effect of *A. dahurica*’s radix water extract (WEAD) on osteoclast differentiation. In vitro experiments showed that WEAD effectively suppresses osteoclast differentiation. Treatment of an osteoclast precursor with WEAD significantly suppressed the expression of nuclear factor of activated T-cells 1 (NFATc1), essential transcription factor for osteoclastogenesis, while increasing the expression of negative regulators, interferon regulatory factor 8 (Irf8) and v-maf musculoaponeurotic fibrosarcoma oncogene homolog B (MafB). Consistent with the in vitro findings, the oral administration of WEAD (100 and 300 mg/kg/day) to mice subjected to surgical ovariectomy for a duration of six weeks alleviated bone loss, while also mitigating weight gain and liver fat accumulation. In addition, we also identified phytochemicals present in WEAD, known to regulate osteoclastogenesis and/or bone loss. These results suggest the potential use of WEAD for treating various bone disorders caused by excessive bone resorption.

## 1. Introduction

Bones are hard tissues that constitute the human body, playing a crucial role in safeguarding soft organs, storing minerals, growth factors and facilitating hematopoiesis [1]. Maintaining bone health is essential for preserving these vital functions, which requires a delicate balance in bone remodeling, encompassing the resorption of old or damaged bone tissue and the formation of new bone [2,3]. However, if this equilibrium is disrupted by factors such as aging or hormonal imbalances, it may lead to bone disorders, including osteoporosis and osteosclerosis [2,4].

The cells responsible for bone remodeling are known to be osteoblasts, which build bones, osteoclasts, which absorb bones, and osteocytes, which coordinate these two cells within the bone [2,3,4]. In particular, the efficient differentiation of osteoclast precursors into multinucleated giant cells (MNCs) is crucial for effective bone absorption. The differentiation process of osteoclasts is initiated by the receptor activator of NF-κB ligand (RANKL), a cytokine secreted by osteoblasts or osteocytes [5]. RANKL binds to its receptor RANK, recruiting TNF receptor-associated factor 6 (TRAF6) to the cytoplasmic domain of RANK, thereby inducing TRAF6 activation [6]. Activated TRAF6 initiates diverse downstream signaling pathways such as NF-κB and mitogen-activated protein kinases (MAPKs). These RANKL/RANK downstream signaling pathways culminate in the expression and activation of the nuclear factor of activated T-cells cytoplasmic 1 (NFATc1), a key transcription factor for osteoclast differentiation [7]. Activated NFATc1 increases the expression of various genes essential for osteoclast differentiation and activity, such as dendritic cell-specific transmembrane protein (DC-STAMP) [8], vacuolar ATPase V_0_ domain d2 isoform (Atp6v0d2) [9] and cathepsin K (CtsK) [10]. In addition, the RANK/RANKL signaling pathway decreases the expression of genes that inhibit osteoclast differentiation, such as interferon regulatory factor 8 (Irf8) [11] and v-maf musculoaponeurotic fibrosarcoma oncogene homolog B (MafB) [12]. Because osteoclast differentiation and bone resorption play a critical role in bone remodeling, there is significant research focused on resolving bone disorders through the regulation of osteoclast differentiation and bone resorption.

Various common foods, such as fruits, soy, garlic and onions, have been reported to exert a positive influence on bones in pharmacological and epidemiological studies [13]. Many cultures, including those in China, Korea and India, have traditional remedies that incorporate herbs and spices for the management of osteoporosis, and some of these natural products are supported by pharmacological evidence [13,14]. Thorough and comprehensive research may justify the exploration of natural products for their potential development into dietary supplements or therapeutic drugs. *Angelica dahurica* radix is a traditional medicinal ingredient that has been used in Korea and China for over a thousand years [15,16]. According to current pharmacological studies, *A*. *dahurica*’s root has various biological activities, including anti-inflammatory [17], antioxidant [18], anticancer [19], antiviral [20] and hepatoprotective effects [21]. Moreover, numerous chemical compounds have been identified in *A*. *dahurica*, with coumarins and volatile oils reported as major active constituents [16]. Despite extensive research on its various properties, there remains limited investigation into the potential impact of *A*. *dahurica*’s root on bone diseases. Therefore, in this study, we investigated the biological activities of *A*. *dahurica*’s radix water extract (WEAD) on bone using a representative osteoporosis animal model, the bilateral ovariectomized (OVX) mouse model [22,23] and bone marrow-derived macrophages (BMMs) as osteoclast precursors.

## 2. Results

### 2.1. WEAD Suppresses Osteoclast Differentiation in BMMs-MLO-Y4 Cells Co-Culture Condition

**Figure 1:**
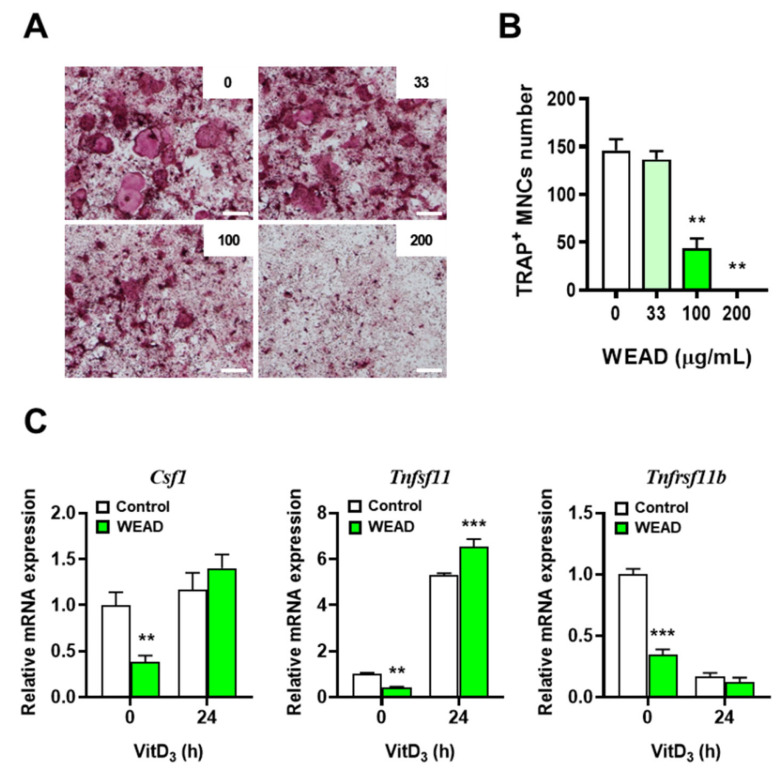
Effect of WEAD on osteogenesis in MLO-Y4/BMMs co-culture. MLO-Y4 and BMMs were co-cultured in the presence of VitD_3_ with diverse concentration of WEAD. (**A**) Microscope images of TRAP-staining (scale bar, 200 µm). (**B**) The number of TRAP-stained MNCs. (**C**) MLO-Y4 were stimulated with VitD_3_ for 24 h. The mRNA levels of important cytokines for osteogenesis were analyzed by real-time PCR. ** *p* < 0.01, *** *p* < 0.001 vs. control without WEAD treatment.

Based on the report that osteoclast differentiation can be induced by RANKL secreted from osteocytes stimulated with 1α,25-dihydroxyvitamin D_3_ (VitD_3_) [24], we evaluated the effect of WEAD on osteoclast differentiation in a co-culture of MLO-Y4 osteocyte-like cells and BMMs. Through tartrate-resistant acid phosphatase (TRAP) staining, a specific staining method for osteoclasts, the results revealed that the osteoclast differentiation decreased in a concentration-dependent manner with WEAD (Figure 1A,B). To determine whether the decreased osteoclast formation was attributed to the action of WEAD on MLO-Y4, we analyzed the changes in the important cytokines involved in osteoclastogenesis expressed in MLO-Y4. As shown in Figure 1C, WEAD did not affect the mRNA expression levels of macrophage colony-stimulating factor (M-CSF, encoded by Csf1) and the decoy receptor for RANKL osteoprotegerin (OPG, encoded by Tnfrsf11b) in MLO-Y4 cells stimulated with VitD_3_ for 24 h. However, the mRNA expression level of RANKL (encoded by Tnfsf11) was further increased by WEAD. Despite the increased mRNA expression of RANKL in WEAD-treated MLO-Y4 cells, osteoclast formation was obviously reduced, indicating that the inhibitory effect of WEAD on osteoclast differentiation may not be attributed to its action on MLO-Y4 cells.

### 2.2. WEAD Inhibits the Differentiation of Osteoclast Precursors

**Figure 2:**
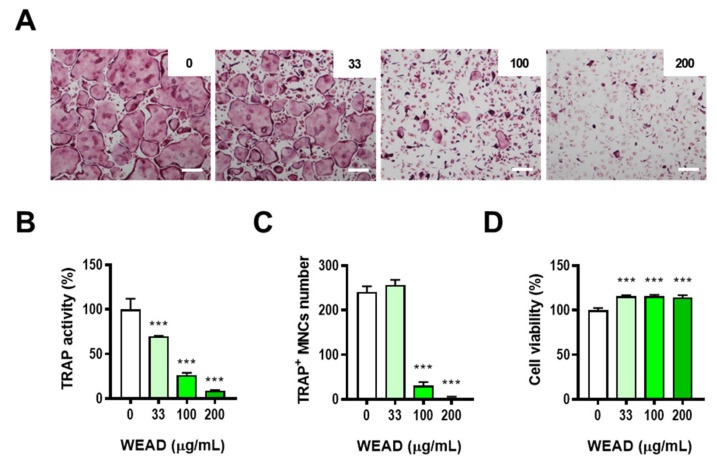
Effect of WEAD on osteoclast differentiation. (**A**–**C**) BMMs were cultured in the presence of M-CSF and RANKL with WEAD (0–200 μg/mL). (**A**) Microscope images of TRAP-staining (scale bar, 200 µm). (**B**) Total TRAP activity. (**C**) The number of TRAP+ MNCs. (**D**) BMMs were treated with WEAD in the presence of M-CSF 24 h. Cell viability was analyzed by CCK-8 kit. *** *p* < 0.001 vs. control without WEAD treatment.

To further investigate whether WEAD has a direct impact on osteoclast progenitor cells, we induced osteoclast differentiation in BMMs by treating them with or without RANKL and various concentrations of WEAD. As a result, TRAP activity and the number of TRAP-stained MNCs were reduced dose-dependently in WEAD-treated BMMs (Figure 2A–C). However, no significant cytotoxicity was observed in BMMs treated with WEAD (Figure 2D). The results from the co-culture and BMMs experiments collectively suggest that WEAD directly inhibits the differentiation of osteoclast precursors, rather than influencing the supporting capacity of osteocytes.

### 2.3. WEAD Disrupts Osteoclastogenic Signal Transduction

**Figure 3:**
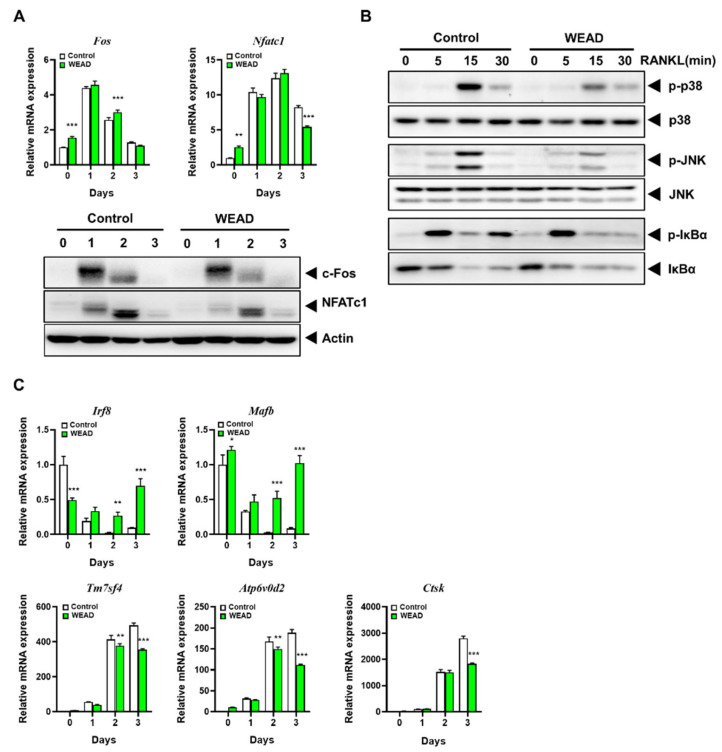
Effects of WEAD on RANK signaling pathways in BMMs. (**A**) BMMs were treated with or without WEAD (200 μg/mL) and RANKL for the specified durations. The mRNA and protein levels of c-Fos and NFATc1 were assessed by real-time PCR and Western blotting, respectively. (**B**) BMMs were pre-treated with WEAD or vehicle for 3 h and then stimulated RANKL for the indicated time. Phosphorylated and non-phosphorylated forms of p38, JNK and IκBα were detected using Western blotting. (**C**) The mRNA expression of *Irf8*, *MafB*, *Tm7sf4*, *Atp6v0d2* and *CtsK*. ** *p* < 0.01, *** *p* < 0.001 vs. vehicle control.

To investigate the mechanism behind the inhibitory effect of WEAD on osteoclast formation, we analyzed the expression of two transcription factors, c-Fos and NFATc1, which play a crucial role in osteoclast differentiation [7,25]. c-Fos, known to regulate NFATc1 expression [26], did not show a decrease in mRNA and protein expression by WEAD treatment, while NFATc1’s mRNA and protein levels decreased (Figure 3A). Among RANK downstream signaling pathways, MAPKs and NF-κB pathways are involved in NFATc1 expression [27]. Therefore, we conducted Western blot analysis to examine whether WEAD affects these signaling pathways. WEAD inhibited the activation of p38 and Jun N-terminal kinase (JNK) MAPKs, while it had no effect on the phosphorylation and degradation of IκBα, a classical component of the NF-κB pathway (Figure 3B). Based on the previous reports [11,12] indicating that Irf8 and MafB function as transcriptional repressors for NFATc1, we investigated whether WEAD affects these two genes during osteoclast differentiation using real-time polymerase chain reaction (PCR). WEAD effectively inhibited the RANKL-induced suppression of these two negative regulators for NFATc1 (Figure 3C). In accordance with NFATc1 reduction, WEAD suppressed RANKL-induced mRNA expression of DC-STAMP (encoded by *Tm7sf4*) and ATP6v0d2, known to be crucial for osteoclast fusion, as well as mRNA expression of CtsK, involved in bone resorption activity (Figure 3C). These findings imply that WEAD hinders osteoclast differentiation by disrupting the RANK signaling pathways that regulate the expression of the positive transcription factor NFATc1 and the negative regulators Irf8 and MafB.

### 2.4. WEAD Ameliorates Bone Loss in OVX Mice

**Figure 4:**
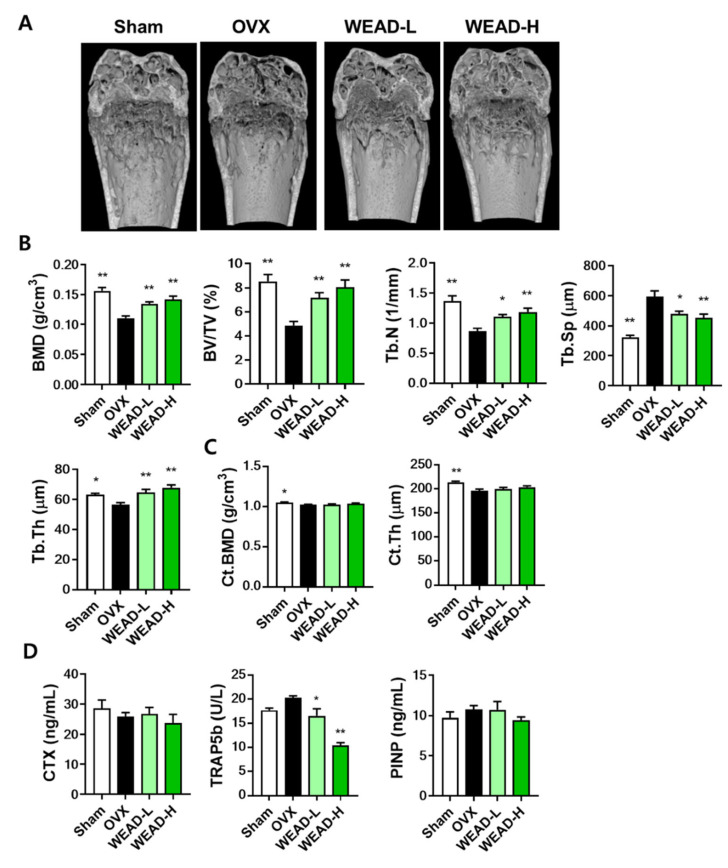
Impact of WEAD on bone loss caused by estrogen deficiency. One week after ovariectomy, WEAD was administered at 100 mg/kg/day (WEAD-L) and 300 mg/kg/day (WEAD-H) for 6 weeks. (**A**) Representative images of distal femur scanned by μ-CT. (**B**) Quantitative analysis of trabecular bone parameters (BMD, BV/TV, Tb.N, Tb.Sp, and Tb.Th). (**C**) Quantitative analysis of Ct.BMD and Ct.Th. (**D**) Measurement of serum levels of CTX, TRAP5b and PINP. * *p* < 0.05, ** *p* < 0.01 vs. OVX control.

Given the inhibitory effects of WEAD on osteoclast differentiation in vitro, we proceeded to assess its therapeutic potential in vivo using a representative osteoporosis model, OVX mice. Prior research has shown that the oral administration of *A. dahurica* water extract, comparable to our WEAD formulation, produced analgesic effects in a mouse model with chronic inflammatory pain at dosages of 100 and 600 mg/kg/day. Notably, the effect was more pronounced at the 100 mg/kg/day dosage [28]. Considering these findings, we examined the impact of WEAD on osteoporotic bone loss using dosages of 100 mg/kg/day (referred to WEAD-L) and 300 mg/kg/day (referred to WEAD-H). Mice were orally administered WEAD for 6 weeks after ovariectomy. To examine microstructure of bones, we conducted micro-computed tomography (μ-CT) imaging and analysis of the distal femur. The μ-CT images displayed evident damage to trabecular bone in the OVX group, while the WEAD-L and WEAD-H groups exhibited a noticeable mitigation of bone loss (Figure 4A). A quantitative analysis of trabecular bone microstructure showed that compared to the OVX group, both WEAD-L and WEAD-H led to a significant increase in bone mineral density (BMD) by 21% and 28%, bone volume per tissue volume (BV/TV) by 47% and 65%, trabecular number (Tb.N) by 27% and 37% and trabecular thickness (Tb.Th) by 14% and 19%. Furthermore, they exhibited a reduction in trabecular separation (Tb.Sp) by 20% and 24%, respectively (Figure 4B). In contrast to the pronounced trabecular bone loss, cortical BMD (Ct.BMD) and cortical thickness (Ct.Th) displayed a slight reduction in the OVX group. The administration of WEAD appeared to mitigate this reduction, although the effect did not reach statistical significance (Figure 4C). These changes in bone parameters suggest the potential beneficial influence of WEAD in mitigating trabecular bone loss. Additionally, we measured the serum concentrations of C-terminal cross-linked telopeptides of type I collagen (CTX, indicative of bone resorption), TRAP isoform 5b (TRAP5b, representing the number of osteoclasts) and procollagen type I N-terminal propeptide (PINP, indicative of bone formation). While there were no significant differences in CTX and PINP levels, WEAD notably decreased TRAP5b levels in comparison to the OVX group (Figure 4D).

**Figure 5:**
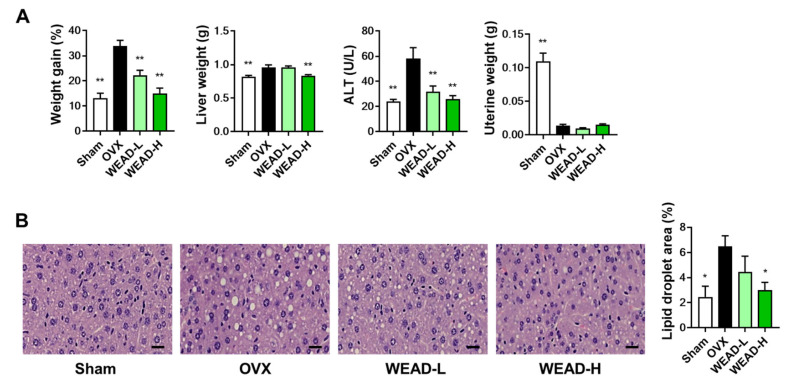
Impact of WEAD on weight gain and liver fat accumulation induced by ovariectomy. (**A**) Assessment of body weight gain, liver weight, ALT levels and uterine weight. (**B**) Microscopic liver images stained with hematoxylin and eosin (scale bar, 20 µm), and quantification of lipid droplets within the images. * *p* < 0.05, ** *p* < 0.01 versus OVX group.

There have been reports suggesting that estrogen deficiency disrupts the balance of energy metabolism, leading to fat accumulation and liver damage [29,30,31]. In particular, the abnormal increase in bone marrow adipose tissue plays a significant role in bone loss in postmenopausal women and OVX models since both osteoblasts and bone marrow adipocytes originate from a common ancestor lineage. [31]. Similarly, as shown in Figure 5, we observed a significant increase in body weight, alteration in liver weight, a substantial rise in alanine aminotransferase (ALT, an indicator of liver injury) levels and fat accumulation in the liver in OVX mice compared to the Sham group. However, we observed that WEAD effectively ameliorated weight gain and fat accumulation in the liver induced by ovariectomy, without exhibiting estrogenic effects, as supported by the absence of changes in uterine weight. These findings indicate that WEAD not only possesses anti-osteoporotic properties but also confers advantageous effects in alleviating liver damage and fat accumulation. However, it remains challenging to establish a certain association between the dual benefits of WEAD. Therefore, to elucidate it, further studies, such as investigations into the effects of WEAD on bone marrow tissue, are essential.

### 2.5. Phytochemical Constituents of WEAD

**Figure 6:**
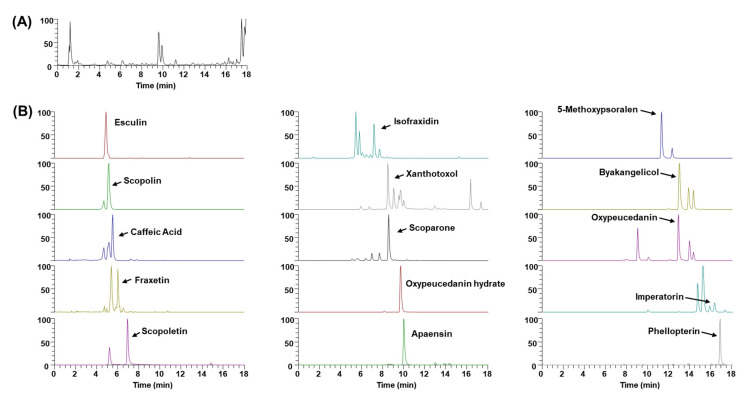
UHPLC-MS/MS analysis of WEAD. (**A**) Base peak chromatogram. (**B**) Extracted ion chromatogram of identified phytochemicals. 1, esculin; 2, scopolin; 3, caffeic acid; 4, fraxetin; 5, scopoletin; 6, isofraxidin; 7, xanthotoxol; 8, scoparone; 9, oxypeucedanin hydrate; 10, apaensin; 11, methoxsalen; 12, byakangelicol; 13, oxypeucedanin; 14, imperatorin; 15, phellopterin.

Having confirmed WEAD’s effectiveness in mitigating bone loss, weight gain and fatty liver associated with estrogen deficiency, we conducted ultrahigh-performance liquid chromatography–tandem mass spectrometry (UHPLC-MS/MS) analysis to characterize the phytochemical profile of WEAD and identify its active components. Based on the mass spectra and retention times, WEAD was identified to comprise one hydroxycinnamic acid (caffeic acid) along with fourteen coumarins: esculin, scopolin, fraxetin, scopoletin, isofraxidin, xanthotoxol, scoparone, oxypeucedanin hydrate, apaensin, methoxsalen, byakangelicol, oxypeucedanin, imperatorin and phellopterin (Figure 6 and Table 1). Among these, esculin [32,33], scopoline [34,35], caffeic acid [36,37], fraxetin [38,39], scopoletin [40,41], isofraxidin [42,43], scoparone [44,45] and imperatorin [46,47] have been previously reported to exhibit hepatoprotective effects, along with inhibitory effects on osteoclastogenesis or anti-osteoporotic effects. Furthermore, bergapten has been demonstrated to hinder osteoclast differentiation and mitigate bone loss in OVX mice [48], while oxypeucedanin and phellopterin have exhibited anti-obesity properties [49]. Therefore, the principal active compounds accountable for the bone-protective and hepatoprotective effects of WEAD encompass esculin, scopoline, caffeic acid, fraxetin, scopoletin, isofraxidin, scoparone, imperatorin, bergapten, oxypeucedanin and phellopterin. It is plausible that the combined actions of these diverse bioactive compounds, each contributing beneficial effects, synergistically underlie the observed protective outcomes of WEAD on both bone and liver.

## 3. Discussion

In the context of drug development, natural products serve as valuable starting points to identify potential drug candidates [50]. Due to their historical use in traditional medicine and empirical knowledge, their pharmacological properties and safety profiles are relatively established. Thorough biochemical and pharmacological studies are essential to uncover the potential therapeutic efficacy of natural products. In our study, we elucidated the pharmacological properties and mechanisms of WEAD.

Osteocytes react to VitD_3_ stimulation by promoting RNAKL expression, while concurrently suppressing the secretion of OPG, thereby regulating osteoclast differentiation [2,24]. In this study, WEAD exhibited a slight augmentation in the increased RANKL expression in VitD_3_-stimulated MLO-Y4 cells. Nonetheless, as demonstrated by the BMMs/MLO-Y4 co-culture outcomes, WEAD effectively hindered osteoclast differentiation. This suppressive influence remained consistent even when treating BMMs with RANKL independently of MLO-Y4, indicating that WEAD could potentially exert a more direct effect on BMMs as opposed to MLO-Y4.

To elucidate the molecular mechanisms behind the pharmacological characteristic of WEAD in inhibiting osteoclast differentiation in vitro in this study, we initially investigated positive factors for osteoclastogenesis. MAPKs and NF-κB pathways induce c-Fos expression, and it binds to the promoter region of NFATc1. The expressed NFATc1 then transcribes key genes crucial for osteoclast differentiation and undergoes self-amplification by binding to its own promoter region, further enhancing osteoclast differentiation. Shown in the results from Figure 3A,B, WEAD suppressed the activation of p38 and JNK, and reduced the expression of NFATc1. This may contribute in part to the inhibitory effect of WEAD on osteoclast differentiation. Furthermore, WEAD influenced not only the positive factors of osteoclast differentiation but also the negative ones. Among studies investigating the negative regulators of osteoclast differentiation, there are reports indicating that Irf8 interacts with NFATc1, inhibiting its transcriptional activity and preventing the expression of its target genes, including its autoregulation [11]. Another study [12] suggested that MafB, in the absence of RANKL stimulation, does not affect the expression of c-Fos while interacting with it to inhibit its binding to the promoter of NFATc1. However, upon RANKL stimulation, MafB is suppressed by the activated p38 and JNK MAPKs, thus releasing c-Fos to induce the expression of NFATc1. In the present study, WEAD increased the expression of Irf8 and MafB, while decreasing the expression of the target genes of NFATc1, including DC-STAMP, ATP6v0d2 and CtsK, indicating that WEAD exerts an inhibitory effect on osteoclast differentiation by affecting both positive and negative regulators.

Furthermore, this study demonstrated the phytotherapeutic activity of WEAD, which exhibited both bone-protective and hepatoprotective effects using the OVX mouse model, an appropriate experimental model for evaluating menopause-associated disorders. In our OVX experiments, WEAD was observed to reduce serum TRAP5b levels. However, there was no significant correlation observed between CTX and PINP levels and alterations in the trabecular bone microarchitecture. This suggests the necessity for more comprehensive studies, including histological evaluation of osteoclasts and osteoblasts, to fully understand the specific mechanisms responsible for WEAD’s bone-protective properties.

While the association between osteoporosis and non-alcoholic fatty liver disease (NAFLD) requires further investigation, pharmacological considerations already exist [51]. There are some treatment options that should be avoided for patients with both conditions. Some medications for NAFLD can increase the risk of bone fractures, while those for osteoporosis can exacerbate nonalcoholic steatohepatitis. Given the widespread occurrence of NAFLD and osteoporosis, addressing these constraints becomes imperative. Our findings indicate that WEAD could be a promising option to broaden the range of drug choices for both osteoporosis and NAFLD.

In summary, this study has revealed the potential of WEAD in bone protection. It demonstrated WEAD’s ability to inhibit osteoclast differentiation in vitro, its bone-protective and hepatoprotective effects in the OVX mice model and identified its phytochemical constituents. However, several uncertainties remain, such as the stability of WEAD, the specific targets and mechanisms underlying the in vivo effects, and more. Further investigations to address these remaining questions could contribute significantly to the development of WEAD as a potential therapeutic agent or dietary supplement for a range of pathological bone conditions.

## 4. Materials and Methods

### 4.1. Extraction of WEAD

The National Development Institute of Korean Medicine (Gyeongsan, Republic of Korea) provided WEAD. It was stored in the herbarium (voucher number #JW72) of the KM Convergence Research at the Korea Institute of Oriental Medicine. After air-drying the roots of *A. dahurica* (0.5 g), they were extracted with 3.5 L of distilled water under reflux for 3 h. The extracts were then filtered and lyophilized to obtain a powder. Prior to use, the powder was dissolved in distilled water and filtered through a 0.2 μm filter.

### 4.2. Materials

Recombinant RANKL and M-CSF were supplied as previously described [52,53]. Acetonitrile and formic acid were obtained from Thermo Fisher Scientific (Waltham, MA, USA). Antibodies against phosphorylated forms including p-p38 (T180/Y182), p-JNK (T183/Y185), p-IκBα (S32) and their non-phosphorylated forms were purchased from Cell Signaling Technology (Danvers, MA, USA). The other antibodies (NFATc1, c-Fos and secondary antibodies) were purchased from Santa Cruz Biotechnology (Dallas, TX, USA).

### 4.3. BMM Isolation and Cell Viability Assay

Bone marrow cells (BMCs) were isolated from the femora and tibiae of 8-week-old C57BL/6J mice following the previously described method [54]. The BMCs were cultured in α-minimal essential medium (α-MEM; HyClone, Logan, UT, USA) supplemented with 10% heat-inactivated fetal bovine serum (FBS; Thermo Fisher Scientific, Waltham, MA, USA) and 20 ng/mL of M-CSF. After one day, non-adherent BMCs were collected and seeded on culture dishes for suspension cells in α-MEM containing 10% FBS and 60 ng/mL of M-CSF. Adherent cells were considered as BMMs after 3 days of culture. To assess the cytotoxicity of WEAD, BMMs were cultured with WEAD for 24 h and analyzed using the Cell Counting Kit-8 (Dojindo Molecular Technologies, Rockville, MD, USA).

### 4.4. Osteoclast Differentiation Assay

BMMs (4 × 10^4^ cells/well), osteoclast precursors, were co-cultured with MLO-Y4 cells (1 × 10^3^ cells/well), an osteocyte-like cell line in α-MEM medium containing 10% FBS in 96-well culture plates. The co-cultures were cultured for 5 days with or without WEAD in the presence of 10 nM of VitD_3_. In order to induce the differentiation of osteoclast precursors, BMMs (1 × 10^4^ cells/well) were cultured in α-MEM supplemented with 10% FBS, 60 ng/mL of M-CSF and 50 ng/mL of RANKL in 96-well culture plates. The BMMs were treated with or without WEAD for 4 days. All cultures were replenished with fresh medium containing supplements on the third day. Subsequently, after fixation with 10% formalin, the cells underwent measurement of TRAP activity and TRAP staining, following the methods described in a prior study [55].

### 4.5. Western Blot Analysis

Cells were lysed using a lysis buffer (PRO-PREPTM; iNtRON Biotechnology, Sung-nam, Korea) for protein extraction. Protein quantification of the collected samples was carried out using a bicinchoninic acid assay kit (Thermo Fisher Scientific). The samples were heated at 100 °C for 5 min to denature them. After that, the samples underwent sodium dodecyl sulfate-polyacrylamide gel electrophoresis for separation. Subsequently, the samples were transferred onto polyvinylidene fluoride membranes (Bio-Rad Laboratories, Hercules, CA, USA) from polyacrylamide gels using a transfer apparatus. The transferred membranes are incubated for 2 h in a shaking incubator with 5% skim milk in TBS-T (150 mM NaCl, 50 mM Tris, pH 7.6 and 0.1% Tween-20) to block them. The membranes were subjected to overnight incubation at 4 °C with a 1:1000 dilution of the primary antibody. The Horseradish peroxidase-conjugated IgG was used as a secondary antibody at a dilution of 1:5000. The detection of the target proteins was performed using a ChemiDoc Touch imaging system (Bio-Rad Laboratories).

### 4.6. Quantitative Real-Time PCR

Total RNA was prepared using the RNeasy Mini kit (Qiagen, Hilden, Germany) following the manufacturer’s instructions. Afterwards, 2 μg of total RNA was used to synthesize cDNA with a high-capacity cDNA reverse transcription kit (Applied Biosystems, Foster City, CA, USA). All TaqMan gene expression assays including c-Fos, (Mm00487425_m1), NFATc1 (Mm00479445_m1), DC-STAMP (Tm7sf4, Mm01168058_m1), Atp6v0d2 (Mm00656638_m1), Ctsk (Mm00484036_m1), Irf8 (Mm00492567_m1), MafB (Mm00627481_s1), M-CSF (Csf1, Mm00432686_m1), OPG (Tnfrsf11b, Mm00435454_m1), RANKL (Tnfsf11, Mm00441908_m1) and 18S rRNA (18s, Hs99999901_s1) were designed by Applied Biosystems. The amplification of the target genes was performed by TaqMan Universal Master Mix II at Quant Studio 6 Flex real-time PCR system (Applied Biosystems). All PCR data were normalized to 18S rRNA as the endogenous control. Relative quantification was calculated using the ∆∆Ct method.

### 4.7. UHPLC-MS/MS Analysis

Authentic standards of phytochemicals identified in *A. dahurica* were purchased from Targetmol (Wellesley Hills, MA, USA). To determine the constituents of *Angelica dahurica*, we performed a UHPLC-MS/MS analysis as previously reported methods [56,57]. Briefly, a Dionex UltiMate 3000 system coupled with a Thermo Q-Exactive mass spectrometer was employed using an Acquity BEH C18 column (100 × 2.1 mm, 1.7 μm) with acetonitrile and 0.1% formic acid in water. The Q-Exactive mass spectrometer was operated in the positive ion modes. Data acquisition and analysis were performed using Xcalibur and TraceFinder (Thermo Fisher Scientific, Waltham, MA, USA).

### 4.8. Animal Study

Six-week-old female C57BL/6J mice were provided by SLC Inc. (Shizuoka, Japan) and housed in a specific pathogen-free facility with a controlled temperature (22 ± 2 °C), humidity (55 ± 5%) and a regular 12-h light/dark cycle. After a one-week acclimatization period in the facility, the mice underwent either ovariectomy or sham surgery. The mice were then divided into four groups, each consisting of six mice: sham, OVX, OVX mice administered with WEAD 100 mg/kg (WEAD-L) and OVX mice administered with WEAD 300 mg/kg (WEAD-H). Starting from one week after the OVX surgery, WEAD was orally administered to the mice once daily, and the standard chow diet was replaced with a purified rodent diet containing 10 kcal% fat (D12450B, Research Diets, New Brunswick, NJ, USA). After the 6-week administration period, the mice were sacrificed following a 7-h fasting period to prepare the samples. The serum levels of CTX, TRAP5b and PINP were measured following the manufacturer’s instructions (Immunodiagnostic Systems Ltd., London, UK).

### 4.9. μ-CT Analysis

The distal femur of the mice was scanned using μ-CT (SkyScan 1276, Bruker, Kontich, Belgium) with an X-ray source operating at 85 kV and 47 μA, resulting in a resolution of 2016 × 1344. Each pixel of the scanned images had a size of 8 μm, and a total of 1100 images from one femur were reconstructed using SkyScan NRecon (version 1.7.42, Bruker). The analysis region for trabecular bone extended from 80 μm below the growth plate to a total length of 1.2 mm in the proximal direction, and it was evaluated using Sky-Scan CTAn (version 1.20.3.0, Bruker). Trabecular BMD, BV/TV, Tb.N, Tb.Sp and Tb.Th were quantitatively analyzed to assess the trabecular bone microstructure. In the analysis of cortical bone, we assessed Ct.BMD and Ct.Th within the region extending from 2 mm to 4 mm below the growth plate in the proximal direction of the scanned femur.

### 4.10. Histological Analysis

Tissues were washed twice with saline and then fixed in 10% neutral buffered formalin for 2 days at room temperature. The fixed tissues underwent a stepwise dehydration process using a series of ethanol dilution before being embedded in paraffin to form blocks. These paraffin blocks were then sectioned into slices with a thickness of 5 μm, and each tissue section was stained with hematoxylin and eosin. Captured through an optical microscope, images of the stained sections were used to quantify the areas of lipid droplets via the ImageJ software (version 1.52a, National Institutes of Health, Bethesda, MD, USA).

### 4.11. Statistical Analysis

We conducted statistical analyses using GraphPad Prism, version 9 (GraphPad, San Diego, CA, USA). The in vitro experimental results were presented as mean ± standard deviation, while the in vivo experimental results were presented as mean ± standard error of the mean. Between-group differences were assessed using a one-way analysis of variance (ANOVA) followed by the Dunnett’s post hoc test or a two-way ANOVA followed by the Sidak’s post hoc test for adjustment of multiple comparisons.

## 5. Conclusions

In summary, our findings demonstrate that WEAD has a direct inhibitory effect on the differentiation of osteoclast precursor cells, while minimally affecting the supportive functions of osteocytes. The inhibitory mechanism of WEAD on osteoclast differentiation involves the negative regulation of the RANKL-induced activation of p38 and JNK MAPKs and reduction of negative regulators, Irf8 and MafB, collectively contributing to the suppression of NFATc1. In addition, WEAD exhibited promising therapeutic potential by ameliorating bone loss and concurrently ameliorating weight gain and hepatic lipid accumulation in OVX mice, without inducing estrogenic effects. Furthermore, we have identified several potential active compounds within WEAD, including esculin, scopoline, caffeic acid, fraxetin, scopoletin, isofraxidin, scoparone, imperatorin, bergapten, oxypeucedanin and phellopterin. Taken together, these findings emphasize the potential of WEAD as a promising novel therapeutic approach for addressing both postmenopausal osteoporosis and hepatic disorders.

## Figures and Tables

**Table 1:** Phytochemicals profiling of *A. dahurica* by UHPLC-MS/MS.

No.	t_R_ (min)	Precursor Ion (*m/z*)			Elemental Composition	Error (ppm)	MS/MS Fragments (*m*/*z*)	Identification
		Estimated	Calculated	Adduct				
1	1.6	168.1019	168.1018	M+H	C_15_H_16_O_9_	−0.887	150.0913	Esculin *
2	1.9	138.0913	138.0914	M+H	C_16_H_18_O_9_	0.546	138.0914	Scopolin *
3	2.3	152.1070	152.1069	M+H	C_9_H_8_O_4_	−0.571	121.0650	Caffeic acid *
4	2.5	166.1226	166.1227	M+H	C_10_H_8_O_5_	0.153	121.0647	Fraxetin *
5	10.7	193.0495	193.0495	M+H	C_10_H_8_O_4_	−0.447	177.0544, 149.0597	Scopoletin *
6	17.8	261.1121	261.1119	M+H	C_11_H_10_O_5_	−1.006	243.1014, 189.0545	Isofraxidin *
7	12.9	207.0652	207.0651	M+H	C_11_H_6_O_4_	−0.537	175.0389	Xanthotoxol *
8	13.1	261.1121	261.1118	M+H	C_11_H_10_O_4_	−1.356	243.1014, 189.0544	Scoparone *
9	13.7	245.0819	245.0815	M+H	C_16_H_16_O_6_	−1.641	175.0389	Oxypeucedanin hydrate *
10	18.7	285.0769	285.0767	M+H	C_17_H_16_O_6_	−0.666	270.0531	Apaensin
11	21.1	271.0965	271.0962	M+H	C_12_H_8_O_4_	−1.214	203.0337	Methoxsalen *
12	9.1	623.1618	623.1613	M+Na	C_17_H_16_O_6_	−0.713	503.1194, 383.0768, 317.0663	Byakangelicol *
13	10.4	609.1461	609.1454	M+H	C_16_H_14_O_5_	−0.588	301.0346	Oxypeucedanin *
14	10.5	431.0984	431.0979	M+H	C_16_H_14_O_4_	−1.019	311.0559	Imperatorin *
15	10.7	595.1668	595.1664	M+H	C_17_H_16_O_5_	−0.667	287.0559	Phellopterin *

* Compared with the retention time and MS spectral data of an authentic standard.

## Data Availability

All relevant data are within the paper.
